# Research on Multi-View 3D Reconstruction Technology Based on SFM

**DOI:** 10.3390/s22124366

**Published:** 2022-06-09

**Authors:** Lei Gao, Yingbao Zhao, Jingchang Han, Huixian Liu

**Affiliations:** School of Electrical Engineering, Hebei University of Science and Technology, Shijiazhuang 050018, China; gaolei@stu.hebust.edu.cn (L.G.); hbkjdxhjc@sina.com (J.H.); liuhuixian@hebust.edu.cn (H.L.)

**Keywords:** multi-view 3D reconstruction, feature-point detection and matching, sparse reconstruction, a dense reconstruction

## Abstract

Multi-view 3D reconstruction technology is used to restore a 3D model of practical value or required objects from a group of images. This paper designs and implements a set of multi-view 3D reconstruction technology, adopts the fusion method of SIFT and SURF feature-point extraction results, increases the number of feature points, adds proportional constraints to improve the robustness of feature-point matching, and uses RANSAC to eliminate false matching. In the sparse reconstruction stage, the traditional incremental SFM algorithm takes a long time, but the accuracy is high; the traditional global SFM algorithm is fast, but its accuracy is low; aiming at the disadvantages of traditional SFM algorithm, this paper proposes a hybrid SFM algorithm, which avoids the problem of the long time consumption of incremental SFM and the problem of the low precision and poor robustness of global SFM; finally, the MVS algorithm of depth-map fusion is used to complete the dense reconstruction of objects, and the related algorithms are used to complete the surface reconstruction, which makes the reconstruction model more realistic.

## 1. Introduction

Multi-view 3D reconstruction technology skillfully combines imaging and computer vision [1]. It reconstructs a 3D model of a target object through a group of images collected by a camera [2]. At the same time, it can also obtain 3D point-cloud information, position information, camera attitude, and internal and external parameters of the camera, etc. With the success of the structure from motion (SFM) algorithm [3], multi-view 3D reconstruction technology has developed rapidly. With the improvement in camera imaging quality and computing power, the multi-view 3D reconstruction method has a good guarantee of efficiency and reconstruction accuracy [4].

Since the 21st century, with the development in computer-vision technology and the significant improvement in computing power, many SFM and MVS methods have emerged. The SFM algorithm depends on the correctness of feature matching between images. Professor Lowe D G proposed the SIFT feature in 2004 [5]. This feature has scale and rotation invariance, which ensures the accuracy of feature matching between images and is widely used in image matching and image retrieval. With the rise in deep learning, researchers began to seek a method based on deep learning to extract feature points. LFT (learned invariant feature transform) is a method that uses deep learning to extract features, so as to replace manually designed features [6]. In 2017, Schbnberger et al. compared manual-design features with deep-learning features. The results show that the current deep-learning features are quite different in different data sets, which proves that deep-learning features still need a large number of data sets [7]. Therefore, in multi-view 3D reconstruction, although there are a variety of new methods, moving structure restoration is still the most popular method. Furthermore, 3D reconstruction technology based on multi-view images has a wide range of applications. In a digital city, the basic geographic environment information plays an important supporting role, which requires the three-dimensional modeling of the city. In the protection of antiquities and ancient buildings, digital museums are obtained through three-dimensional reconstruction technology. In the driverless field, the car needs to perceive the surrounding environment and a high-precision map, which depends on three-dimensional reconstruction technology. In the game and film industry, it is necessary to make some character models or special effects, which can also be obtained by this method. With the continuous development of multi-view image 3D reconstruction technology, industrial reform and economic development will be promoted.

The traditional structure from motion (SFM) algorithm is divided into incremental SFM and global SFM [7]. Incremental SFM successively calculates the camera parameters and scene structure; the global SFM calculates the parameters and scene structure of all cameras according to the constraint relationship of all cameras [8]. Incremental SFM has high robustness, but it runs for a long time; in large scenarios, drift may occur due to error accumulation [9]. Global SFM runs quickly and will not drift, but the reconstruction accuracy is low and the robustness is not high [10].

Aiming at the problems existing in the traditional incremental and global SFM, this paper designs a hybrid SFM algorithm. Combined with the advantages of incremental and global SFM, the image is divided into multiple subsets. In the subsets, the incremental SFM algorithm is used to recover the camera parameters of the subset, which has high robustness and high precision. Then, the global SFM algorithm is used to calculate the parameters of all cameras, spread the errors to each camera, and triangulate to obtain the sparse model of the scene. Finally, bundle adjustment is performed to optimize camera parameters and the sparse model.

## 2. Principle and Process of 3D Reconstruction

### 2.1. Camera Model

Multi-view 3D reconstruction is mainly divided into four steps: feature-point extraction and matching, sparse point-cloud reconstruction, dense point-cloud reconstruction and surface reconstruction [11,12]. Its main principle is the pinhole camera imaging model based on optics; that is, there is point W in a three-dimensional space and the projection point $W^{\prime }$ on the imaging plane to meet the mathematical model of central photography, as shown in Formula (1):

(1) $$sW^{\prime }=s\left[\begin{array}{c} u\\ v\\ 1 \end{array}\right]=\left[\begin{array}{ccc} f_{x} & \gamma & u_{0}\\ 0 & f_{y} & v_{0}\\ 0 & 0 & 1 \end{array}\right]\left[\begin{array}{c} x_{c}\\ y_{c}\\ z_{c} \end{array}\right]=\left[\begin{array}{ccc} f_{x} & \gamma & u_{0}\\ 0 & f_{y} & v_{0}\\ 0 & 0 & 1 \end{array}\right]\left[\begin{array}{c} R\;t \end{array}\right]\left[\begin{array}{c} x_{w}\\ y_{w}\\ z_{w}\\ 1 \end{array}\right]=K\left[\begin{array}{c} R\;t \end{array}\right]W=\mathrm{PW}$$

where ${\left[x_{w}\;y_{w}\;z_{w}\;1\right]}^{T}$ is the homogeneous coordinate of the three-dimensional point in the world coordinate system [13]; ${\left[u\;v\;1\right]}^{T}$ is the homogeneous coordinate of the point on the two-dimensional physical image plane in the image coordinate system; $f_{x}$, $f_{y}$ are the equivalent focal length in *x* and *y* directions; $u_{0}$, $v_{0}$ are the coordinate of the main point, that is, half of the length and width of the image; γ is the distortion parameters, which together constitute the camera internal parameter K and are only related to the camera structure; R is the camera rotation matrix; and t is the translation matrix [14,15]. R and t together form the camera external parameter matrix, which describes the position relationship between the camera coordinate system and the world coordinate system [16]. P is the projection matrix, and using the simultaneous equations of the matching points of at least two images, the three-dimensional coordinates of the spatial points can be solved [17].

### 2.2. Introduction to SFM Theory

For a static scene, the multi-view image of the scene is used for sparse reconstruction. After extracting and matching the feature points of the image, the camera parameters and scene structure are calculated [18]. The above process is to restore the structure from motion (SFM). Motion refers to the motion trajectory of the camera, that is, the camera parameters, and structure refers to the 3D points of the scene, that is, the sparse model of the scene. The traditional SFM algorithm can be divided into incremental and global according to the order of adding images. The incremental SFM algorithm first selects the initial image pair, then registers the images in turn, and calculates the camera parameters and scene structure. The global SM algorithm calculates all camera parameters and the scene structure according to the global constraint relationship of the camera [19].

### 2.3. Introduction to MVS Theory

After sparse reconstruction of a scene, the camera parameters, sparse 3D points and their corresponding image 2D points are obtained, but this information can not completely represent a scene, so it needs to be transformed into a dense representation of the scene. There are many ways to represent the dense model of the scene, mainly including voxels, depth maps and dense point clouds [20]. Dense reconstruction of scene is also called MVS. MVS captures more scene viewpoints to improve robustness and reduce the impact of image noise and surface texture. It is usually divided into voxel-based algorithms, point-cloud diffusion algorithms and depth-map fusion algorithm, according to the representation of scene [21].

## 3. Design of 3D Reconstruction Algorithm

### 3.1. Extraction and Matching of Image Feature Points

A scale invariant feature transform (SIFT) algorithm is mainly composed of four parts: constructing scale space, extracting key points, assigning main directions and generating feature-point descriptors [22]. The main idea is to filter the extreme points found in the scale space, so as to find the stable feature points. Finally, the local features of the image are extracted around each stable feature point to form a local descriptor and use it in future matching [23].

Speeded up robust features (SURF) is an efficient variant of SIFT. The principles of SURF feature extraction and SIFT feature extraction are consistent, but the method used for SURF is different from SIFT. The determinant value of Hessian matrix is used as the feature detection of SURF, and the integral graph is used to improve the operation efficiency. The descriptor of SURF is based on the response of a 2D discrete wavelet transform and makes efficient use of the integral graph [24], which is more efficient and accurate than SIFT in running speed and brightness change; however, SIFT works better in the case of scale and rotation transformation, so the SIFT feature-extraction results and SURF feature-extraction results are fused to learn from each other and increase the number of matching feature points [20,21].

After generating the feature descriptor, the nearest neighbor matching algorithm (nearest neighbor—NN) of Euclidean distance is used to complete the rough matching. The Euclidean distance measurement formula is shown in (2).


(2) $$\{\begin{array}{c} D_{\mathrm{ssd}}(a,b)=\|a-b{\|}_{2}^{2}=D_{\mathrm{euc}}{\left(a,b\right)}^{2}\\ D_{\mathrm{euc}}(a,b)=\|a-b{\|}^{2}={\left[\sum_{i=1}^{n}(a_{i}-b_{i})\right]}^{\frac{1}{2}} \end{array}$$

The process is as follows: each feature vector in image 1 is represented as a, and a KD tree is used to search in image 2 to find the distance between all feature points in image 2 and a. When the Euclidean distance is less than a threshold, the feature with the smallest distance is taken as the matching feature point. In order to improve the robustness of matching, this paper adds the constraint ratio: nearest neighbor eigenvector $b^{*}$ and next nearest neighbor distance vector $b^{**}$; when the ratio of nearest neighbor distance to next nearest neighbor distance is less than a given threshold, i.e., $D\left(a,b^{*}\right)/D\left(a,b^{**}\right)<\alpha$, then $b^{*}$ is the match of a, otherwise it is considered that a does not match in image 1. In the experiment *α* = 0.7.

After matching the feature points between pictures, there is often a false matching phenomenon. Then, it is necessary to verify or optimize the feature matching. The feature points with false matching are filtered. The false matching can be eliminated by camera pose estimation. The random sample consensus (RANSAC) algorithm is a random iterative process which separates internal points and noise outliers through user-defined threshold size identification. The algorithm uses the smallest possible initial data set to calculate a model, and uses the consistency number to expand this set. The goal of the algorithm is to fit a model on the data set containing outer points. In the process of each iteration, the feature points acceptable to the constraint model and within the error threshold are called inner points. After running the set number of iterations, the constraint function containing the most inner points will be returned.

### 3.2. Traditional SFM

In the sparse reconstruction stage, the traditional SFM algorithm is divided into incremental and global. The incremental SFM successively calculates the camera parameters and scene structure. The global SFM calculates the parameters and scene structure of all cameras according to the constraint relationship of all cameras.

**Figure 1:**
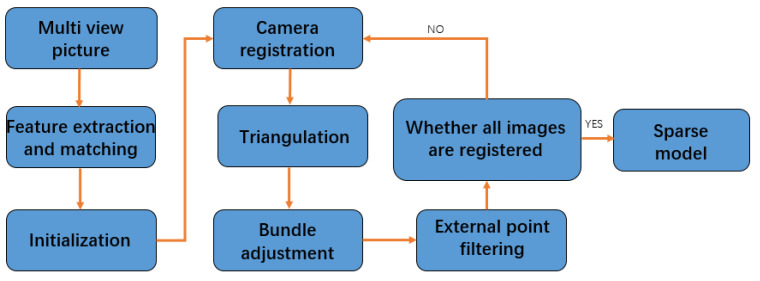
The flow chart of an incremental SFM algorithm.

The flow of incremental SFM is shown in the Figure 1. Firstly, initialize, select a pair of pictures as the initial picture pair, require the image pair to have enough matching points and meet the wide baseline conditions, calculate the rotation and position relationship between its cameras, triangulate the matched feature points to obtain the initial 3D points as the initial model, perform bundle adjustment, and optimize the camera parameters and initial model. Then, add new images, in turn, for registration, according to the corresponding relationship between the 2D points and 3D points in the new image, calculate the parameters of the newly registered camera through a PnP (perspective-n-point) algorithm, triangulate the new feature points, and obtain new 3D points to be added to the original model. In this process, the bundle adjustment is continuously performed to optimize the camera parameters and 3D point coordinates, and the external points are filtered until all images are reconstructed to obtain a sparse 3D model.

The flow chart of an incremental SFM algorithm is shown in the Figure 1.

**Figure 2:**
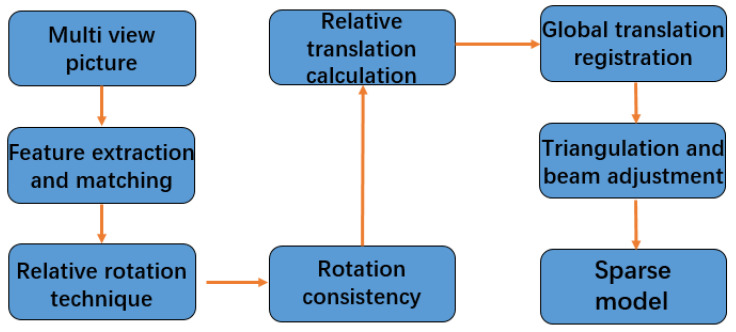
The flow chart of a global SFM algorithm.

The algorithm flow of global SFM is shown in the Figure 2. After obtaining the corresponding relationship of image feature points, first, calculate the relative rotation relationship between cameras, and use the rotation consistency to remove the wrong feature matching, then calculate the translation matrix between cameras through three-view constraints and register it in the global coordinate system to obtain all camera parameters. Finally, triangulate the 3D points corresponding to feature points, and perform a bundle adjustment to optimize camera parameters and scene structure. The sparse 3D model of the scene is obtained.

The flow chart of a global SFM algorithm is shown in Figure 2.

Incremental SFM has high robustness, but it runs for a long time. In the case of large scenes, drift may occur due to error accumulation. Global SFM runs quickly and will not drift, but the reconstruction accuracy is low, and the robustness is not high. Here, robustness refers to the ability to resist external influences, such as lighting, whether the object texture is rich, shooting angle, etc. The higher the robustness, the more it can ignore the influence of these external factors for reconstruction; on the contrary, the reconstruction may fail.

### 3.3. Hybrid SFM

In view of the problems existing in the traditional incremental and global SFM, this paper outlines a hybrid SFM algorithm, which combines the advantages of incremental and global SFM. As shown in the image, the image is divided into multiple subsets: the incremental SFM algorithm is used to recover the camera parameters of the subset in the subset, which has high robustness and high precision. Then, the parameters of all cameras are calculated by using the global SFM algorithm, and the error is spread to each camera. The sparse model of the scene is obtained by triangulation. Finally, the bundle adjustment is performed to optimize the camera parameters and sparse model.

**Figure 3:**
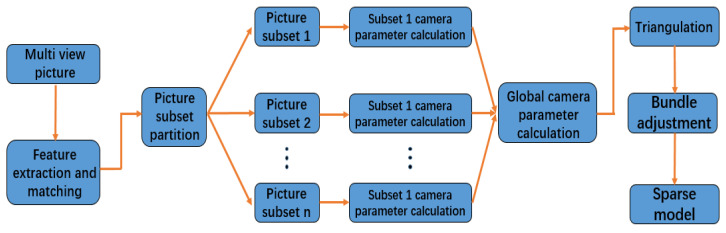
The flow chart of the hybrid SFM algorithm.

The flow chart of the hybrid SFM algorithm proposed in this paper is shown in Figure 3.

#### 3.3.1. Image Subset Division

After the preprocessing of image feature extraction and matching an image set $I=\left\{I_{i}\right\}$, a SIFT feature point set $F=\left\{F_{i}\right\}$ and its corresponding relationship $M=\{M_{ij}|M_{ij}\in F_{i}\times F_{j},j\neq i\}$, where $M_{ij}$ is a set of feature correspondence between two images $I_{i}$ and $I_{j}$, each image $I_{i}$ is associated with a camera. An image set is represented as a camera geometry image G=(V,E), where V and E are sets of vertices and edges. Image clustering is to divide the image into several camera subgraphs $\left\{G_{k}|G_{k}\left(V_{k},E_{k}\right)\right\}$, that is, the image set is divided into multiple subsets, and each image set needs to meet size constraints and integrity constraints.

When the scale of SFM is expanded, due to the limitation of computing power and memory, it is difficult to efficiently reconstruct with incremental or global SFM. Therefore, it is divided into several small sub-problems. The images are clustered to obtain multiple image sets, and there are enough duplicate images between all sets. In order to meet the computational performance and subsequent global camera parameter estimation requirements, each image set needs to meet the size constraints and integrity constraints.

The size constraint requires that the size of each image set is small and similar. Firstly, each image set should be small enough to adapt to the computing resources of the computer and realize efficient incremental SFM calculation. Moreover, the small-scale SFM problem can effectively avoid a lot of time consumption and possible drift caused by continuous beam adjustment.

In order to provide sufficient constraints for the calculation of global camera parameters, we introduce integrity constraints to ensure the connectivity of different subgraph cameras. However, completely preserving the connectivity between cameras will introduce too many duplicate cameras in different sets, and it is difficult to meet the size constraint. Therefore, each camera is defined as a complete set, as shown in the Formula (3).

(3) $$\partial (G_{i})=\frac{\sum_{j\neq 1}|V_{i}\cap V_{j}|}{\left|V_{i}\right|}$$

where $\left|V_{i}\right|$ is the number of images of subset i, and $\left|V_{i}\cap V_{j}\right|$ is the number of identical pictures in subset i and subset j. This quantifies that a camera set $G_{i}$ is covered by other camera sets, which limits the number of duplicate cameras, and ensures that all camera sets have enough overlapping cameras to completely reconstruct the scene with adjacent sets.

In order to meet the size constraints and integrity constraints at the same time, a graph-based camera clustering algorithm is used. The camera subset is obtained by iteratively running graph segmentation and graph expansion. The steps are as follows.

(1) Graph segmentation by recursively dividing the camera geometry image that violates the size constraint into smaller images to meet the size constraint. Starting from the camera image G, the normalized graph cut algorithm is iteratively applied to all subgraphs $G_{i}$ that do not meet the size constraint; $G_{i}$ is divided into two balanced subgraphs $G_{i1}$ and $G_{i2}$ until no subgraph violates the size constraint. Generally, image pairs with a large number of matching features have high edge weight in the graph and are unlikely to be cut.
(2) The graph extension satisfies the integrity constraint by introducing enough overlapping cameras between adjacent camera sets. The cut edges are arranged in descending order of weight $w\left(e_{i}\right)$, and V is added iteratively $V_{i}$ and $V_{j}$ to its associated subgraph $G\left(V_{i}\right)$ or $G(V_{j})$ until the subgraph satisfies the integrity constraint. After adding a small number of relevant vertices of discarded edges, it is not difficult to meet the integrity constraint.

After graph expansion, the size constraint may not be satisfied, so iteration between graph segmentation and graph expansion must be performed; when both constraints are satisfied, the iteration ends.

#### 3.3.2. Subset Camera Parameter Calculation

After the image set is divided into multiple subsets, incremental SFM is used to recover the camera parameters of each subset. Firstly, initialize, select a pair of pictures as the initial images, calculate the rotation and position relationship between their cameras, triangulate the feature points to obtain the initial model, then register a new image in turn and triangulate to obtain new 3D points. In this process, bundle adjustment is continuously performed to optimize the camera parameters and 3D points until all images are registered.

In the process of registering a new image, the perspective-n-point (PnP) algorithm can effectively calculate the parameters of the newly registered camera. This algorithm is a method to solve the camera parameters from the correspondence between 3D points and 2D points, in which 3D points are the coordinates of the scene model in the world coordinate system, and 2D points are the points projected onto the image by these 3D points. Therefore, it is necessary to obtain the rotation and position relationship of the camera coordinate system relative to the world coordinate system, and align the newly registered camera with the existing scene model.

**Figure 4:**
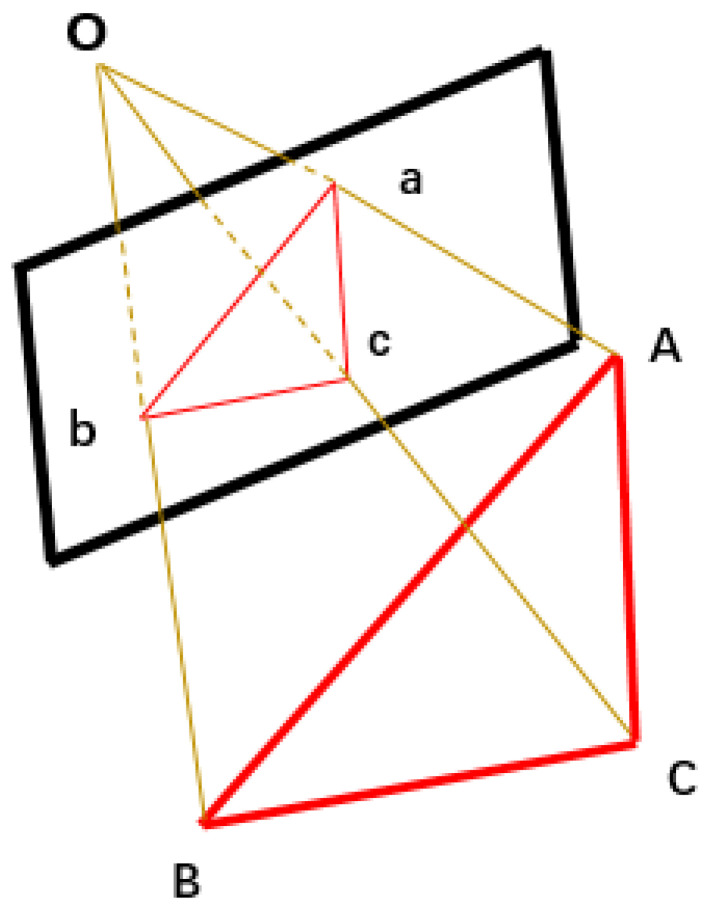
P3P schematic diagram.

When there are three pairs of 3D points and 2D points, it is a P3P problem. As shown in Figure 4, points A, B and C are the 3D points of the scene in the world coordinate system, and points a, b and c are the corresponding 2D points of the image.

Firstly, the coordinates of points A, B and C in the current camera coordinate system are obtained, then the rotation and position parameters of the camera are calculated according to the 3D point coordinates in the world coordinate system and the 3D point coordinates in the current camera coordinate system. According to the mathematical relationship and mathematical formula derivation, we can obtain Formula (4):

(4) $$\{\begin{array}{c} a_{4}x^{4}+a_{3}x^{3}+a_{2}x^{2}+a_{1}x+a_{0}=0\\ b_{1}y-b_{0}=0 \end{array}$$

The equations have four sets of solutions, of which only one is suitable, so an additional 3D point is required for verification. According to the values of x and y, OA, OB and OC can be obtained, and then the coordinates of points A, B and C in the camera coordinate system can be calculated from the camera’s internal parameters. Finally, the rotation and position matrix of the camera are calculated according to the coordinates of points A, B and C in the camera coordinate system and their coordinates in the world coordinate system. At the same time, the selection of a set of appropriate camera external parameters is verified by additional 3D points.

After registering the new camera in the coordinate system of the existing model, the new feature points are triangulated to obtain new 3D points, and the camera parameters and scene model are optimized through bundle adjustment to reduce the re-projection error of the 3D points of the existing model. Finally, the external points are filtered, and the 3D points are connected with their corresponding 2D points. If the maximum included angle is less than 2°, the point is eliminated; the 3D points with excessive re-projection error are also eliminated.

Through the above method, the images are added successively and the camera parameters are restored. When all images are added, the work is ended, so as to obtain the camera parameters of each subset.

#### 3.3.3. Global Camera-Parameter Calculation

After obtaining the camera parameters of each subset, all camera parameters need to be unified into the same coordinate system. The global algorithm calculates the global camera parameters in two steps. First, calculate the global rotation relationship of the camera, and then calculate the global translation relationship of the camera.

The algorithm for calculating the global rotation matrix is designed as follows:

(1) Set the rotation matrix of the first camera to $R_{1}=I$;
(2) Construct global camera rotation equation;
(3) Solve the global camera rotation matrix by least square method;
(4) Obtain the rotation matrix satisfying orthogonality by SVD.

The algorithm for calculating the global translation matrix is designed as follows:

(1) Set the scale of the first image subset to $\alpha_{1}=1$;
(2) Set the position of the first camera to $c_{1}=0_{3\times 1}$;
(3) Construct global camera position equation;
(4) Perform convex optimization for global camera position;
(5) Convert global camera position to global translation matrix.

The detailed design of the algorithm for solving the global rotation matrix and translation matrix is as follows. Firstly, calculate the global rotation matrix of the camera, obtain the accurate relative rotation matrix $R_{ij}$ through the incremental formula, convert the relative rotation into the global rotation $R_{i}$, set the rotation matrix of the first camera to $R_{1}=I$; its relationship is shown in Formula (5).

(5) $$R_{i}=R_{ij}R_{i}$$

Generally, the rotation relationship between cameras is greater than the number of cameras. Formula (5) is an overdetermined equation. However, due to the influence of noise, there is usually no solution that accurately meets the above equation, so this problem is solved by the least square method.

Convert the above problem into three sub-problems, as shown in Equation (6).

(6) $$r_{j}^{k}-R_{ij}r_{j}^{k}=0_{3\times 1}$$

where, $r_{j}^{k}$ is column k of $R_{i}$, k=1,2,3. The three sub-problems are solved by the least square method to obtain the global rotation matrix $R_{i}$. Since the rotation matrix also needs to meet the orthogonality, the appropriate rotation matrix $R_{i}$ is obtained by singular value decomposition (SVD).

Given the global rotation relationship between cameras, their positions are expressed by a linear equation, as shown in Equation (7).

(7) $$\alpha_{k}t_{ij}^{k}=R_{j}\left(c_{i}-c_{j}\right)$$

where $t_{ij}^{k}$ is two cameras $c_{i}$ and $c_{j}$ for the relative translation relationship between both cameras are from the *k*-th camera set, and their scale factor is $\alpha_{k}$. Then, Equation (7) is rewritten as $\alpha_{k}R^{T}t_{ij}^{k}=c_{i}-c_{j}$. The scale factor of all camera sets is expressed as $x_{s}={\left[a_{1},\ldots ,a_{M}\right]}^{T}$, representing all camera positions as $y_{c}={\left[c_{1},\ldots ,c_{N}\right]}^{T}$, and the linear equations are obtained, as shown in Formula (8).

(8) $$\underset{A_{ij}^{k}}{\underset{︸}{\left[\cdots p\cdots \right]}}x_{s}=\underset{B_{ij}}{\underset{︸}{\left[\cdots I\cdots -I\cdots \right]}}y_{c}$$

where, $A_{ij}^{k}$ is a 3×M of the matrix, whose appropriate position is $p=R_{j}^{T}t_{ij}^{k}$, and the rest are $O_{3\times 1}$, $B_{ij}$ is a 3×3N of the matrix, whose appropriate position of matrix is $I_{3\times 3}$ and $-I_{3\times 3}$, the rest is $0_{3\times 3}$, the linear equations of all camera sets are put into one equation to obtain Equation (9).

(9) $$Ax_{s}=By_{c}$$

Setting $c_{1}=0_{3\times 1}$, $\alpha_{1}=1$, the positions of all cameras are obtained by solving the following convex optimization problem.


(10) $$\arg\underset{x_{s},y_{c}}{\min}{\left\|Ax_{s}-By_{c}\right\|}_{1}$$

After obtaining the global parameter relationship of all cameras, for a 3D point, if the number of visible cameras is greater than or equal to 3, the corresponding track is triangulated to obtain the sparse model of the scene. Finally, beam adjustment is performed to optimize the camera parameters and 3D model.

#### 3.3.4. Prediction Results of Hybrid SFM

As the global SFM significantly depends on the robust rotation matrix, only one BA optimization is carried out in the beam adjustment, and the reconstruction speed is very fast, but the sparse reconstruction accuracy is not high; incremental SFM continuously adds images for incremental BA optimization, so the calculated rotation matrix and translation matrix are robust, and the accuracy of sparse model is good. However, due to the accumulated error of incremental multiple BA optimization, scene drift will occur. The hybrid SFM divides the image set into multiple subsets, and incremental SFM is carried out in the subsets, which not only ensures the accuracy of the model, but also avoids the cumulative error caused by adding images many times. After completing the incremental SFM in the subsets, the process of global SFM is carried out, and BA optimization is carried out again to allocate the error to each camera to increase the accuracy of the sparse model.

Here, we assume that, in terms of reconstruction accuracy, the hybrid SFM will show an improvement compared with incremental SFM and global SFM; for large data sets, hybrid SFM will require a shorter reconstruction time than incremental SFM.

### 3.4. Dense Reconstruction

Taking the camera parameters and sparse model obtained by hybrid SFM as input, first, take each image as the reference image, select the neighborhood image to form a stereo image pair, and then calculate the depth map of the reference image to represent a scene. After the depth map is fused, the dense point cloud of the scene is obtained, which is more convenient to observe a scene.

**Figure 5:**
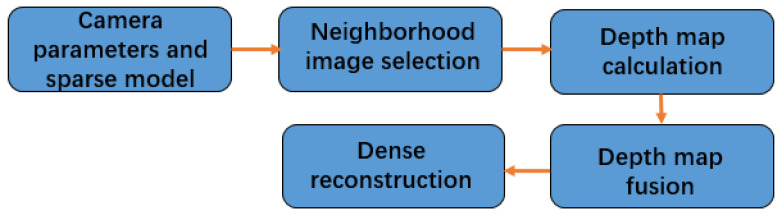
The MVS algorithm based on depth-map fusion.

The MVS algorithm based on depth-map fusion is shown in the Figure 5.

N images are selected as the neighborhood images of each reference image. In order to ensure the consistency and accuracy of dense matching, these images should have sufficient similarity and provide a large enough baseline. The criteria for selecting neighborhood images are the number of matching feature points in the sparse reconstruction process and the angle between the sparse 3D points and the optical center of the two image cameras. It is required that there are as many matching feature points as possible, and the included angle is large. N field images are selected for the reference image to form a stereo image pair, and the depth map of the reference image is calculated.

Calculating the depth map of the reference image includes two parts, a region generation framework and a matching system. The region generation framework maintains a priority queue Q, which stores candidate matching points, including the position, depth and normal vector of features in the reference image. The matching system takes the candidate matching points as the input to optimize the corresponding depth and normal vector.

### 3.5. Surface Reconstruction

In this paper, the floating-scale surface-reconstruction method based on long symbolic distance is used to reconstruct the surface of the dense reconstructed model, and then automatically create the texture image. This algorithm is mature and has been widely used, and the mathematical theory is cumbersome, so it will not be introduced in detail here.

**Figure 6:**
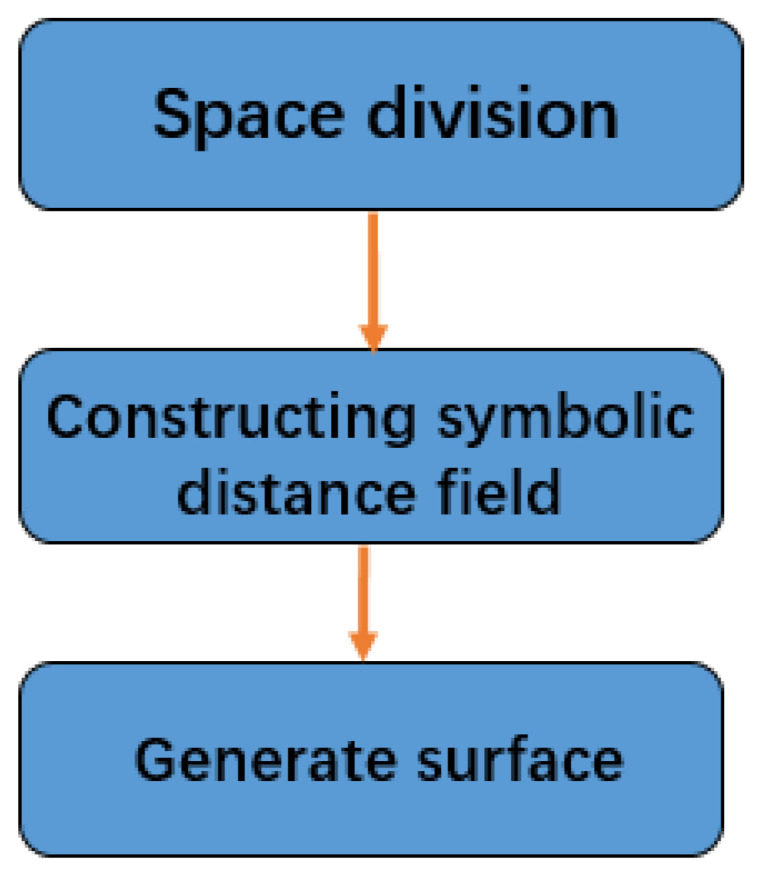
The flow chart of surface reconstruction.

The flow chart of surface reconstruction is shown in Figure 6.

**Figure 7:**
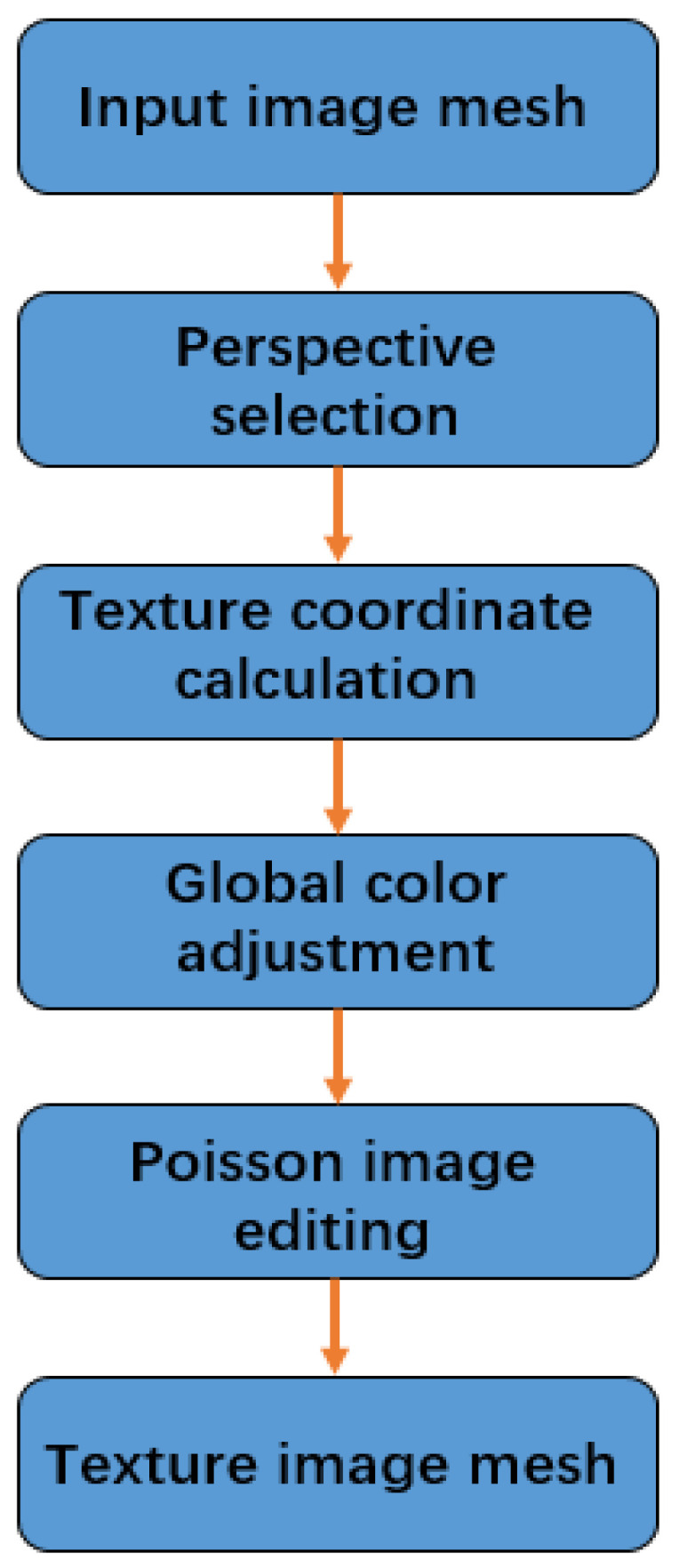
The flow chart of texture-creation algorithm.

The flow chart of the texture-creation algorithm is shown in Figure 7.

## 4. Experimental Verification and Data Analysis

All experiments in this paper were carried out in a ubuntu18.04 64-bit operating system, using open-source frameworks OPENMVG and OPENMVS, using C++ programming language to complete the corresponding algorithm.

An iPhone 11 was used to capture the wallet to obtain the data set “wallet” used in this experiment (a total of 18 pictures were taken in this data set, and the side and front of the scene were taken). In addition to conducting relevant experiments in our own scenes, we also conducted relevant experiments on the official public data set, and the experimental results met the expectations and requirements. Due to copyright issues, the experimental results of public data sets will not be displayed here.

When taking an image, relevant EXIF information was retained in the attribute item; the camera focal length, aperture value, width and height corresponding to image resolution and other information can be obtained. Different cameras correspond to different sizes of *CCD* components, which can be obtained by querying relevant cameras. After obtaining the above information, the pixel information of the focal length can be calculated by the following Formula (14), so as to obtain the internal parameter matrix of the camera:

(11) $$f_{\mathrm{pixel}}=\frac{\max\left\{\left(w_{\mathrm{pixel}},h_{\mathrm{pixel}}\right)\right\}*f_{mm}}{CCD_{mm}}$$

In Equation (11), $w_{\mathrm{pixel}}$, $h_{\mathrm{pixel}}$ are the width and length pixel information in the image resolution information, and $f_{mm}$ is the focal length information in EXIF, $CCD_{mm}$ is the CCD information of the corresponding camera in the exchangeable image file format.

### 4.1. Image Feature Extraction and Matching

**Figure 8:**
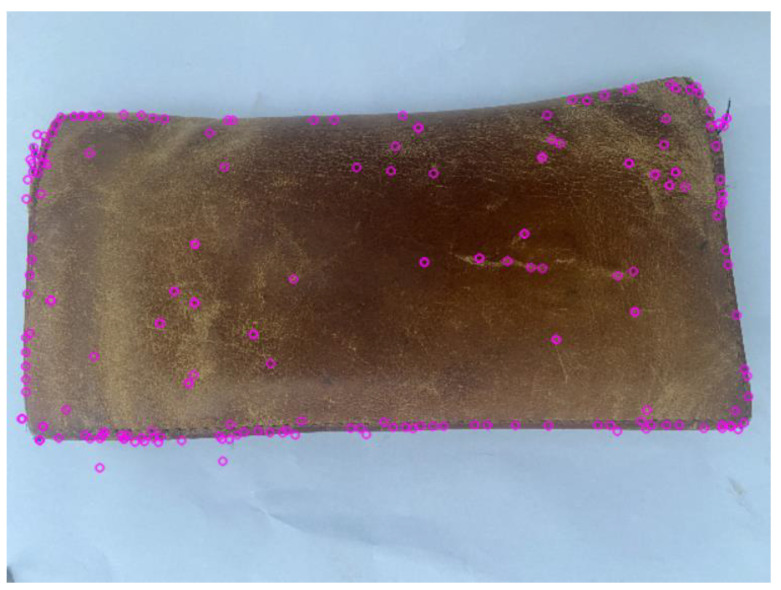
Feature-point extraction.

The feature points of the picture were extracted by matching and fusing the feature extraction results of SIFT and SURF algorithms, as shown in the Figure 8.

Figure 8 shows that all the edge and corner features of the object in the picture are basically extracted, which verifies the efficiency of the feature extraction algorithm in this paper.

After generating the feature descriptor, the nearest neighbor matching algorithm of Euclidean distance was used to complete the rough matching; the constraint ratio was added to improve the robustness of matching. There are often mismatches after feature-point matching between images; RANSAC algorithm was used to eliminate the mismatching.

**Figure 9:**
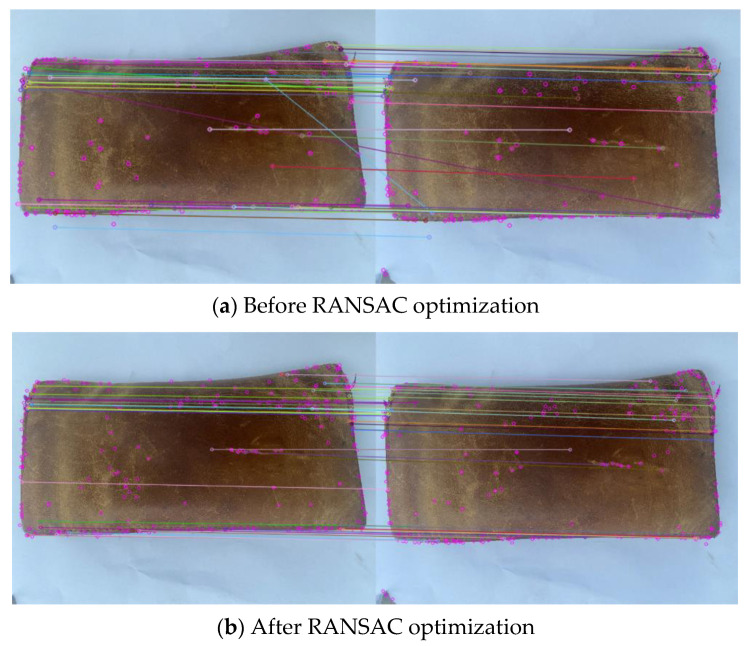
(**a**,**b**) are the experimental diagrams of feature matching before and after RANSAC optimization.

The experimental diagram before and after RANSAC optimization is shown in Figure 9.

As the material of the experimental data set “wallet” is leather, the skin is relatively smooth, greatly affected by light and has less surface-texture information, which cannot fully reflect the effectiveness of the feature-point extraction and matching method proposed in this paper. A set of experimental results are supplemented here.

**Figure 10:**
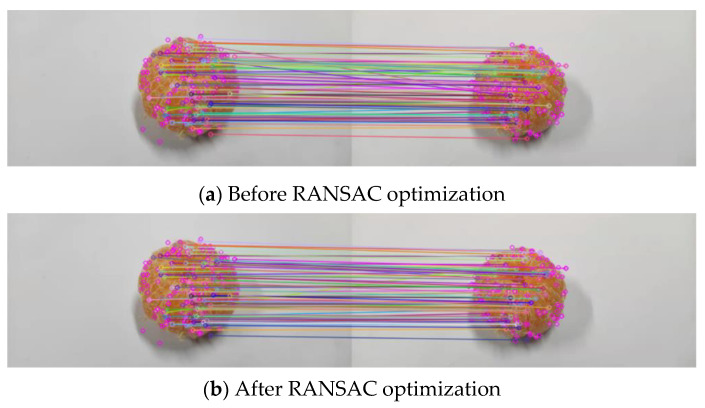
(**a**,**b**) is the supplementary experimental diagram of feature matching before and after RANSAC optimization.

In Figure 9, the number of matching pairs before optimization is 44, and the number of matching pairs after eliminating wrong matching is 40; in Figure 10, the number of matching pairs before optimization is 86, and the number of matching pairs after eliminating false matching is 81. As can be seen from Figure 9 and Figure 10, after adding the constraint proportion, the coarse-matching process can correctly complete most of the feature-point matching, but there are still many incorrect matchings. The incorrect matching can be effectively eliminated by using RANSAC algorithm.

### 4.2. Data Comparison of Three Sparse-Reconstruction Methods

Both hybrid SFM and traditional SFM are based on image feature-point extraction and matching, so as to restore the sparse point cloud of the reconstructed object. The more complex the geometric structure of the reconstructed object is, the more image feature points are obtained, the more matching pairs are, and the better the reconstruction effect is. When acquiring the object image, it is necessary to ensure that there are enough repeated parts for each adjacent two images to match the image pairs. This may be difficult for an object with less structure and texture, because there are fewer feature points extracted from the object, fewer sparse point clouds, or there may even be reconstruction failure.

**Figure 11:**
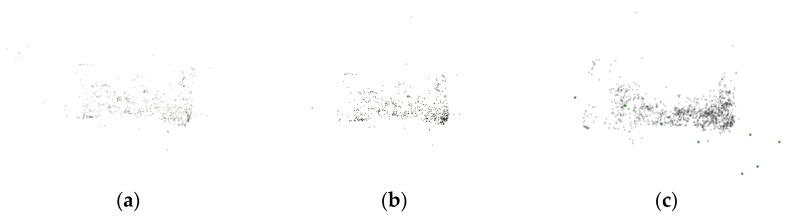
(**a**–**c**) are sparse models reconstructed using global, incremental and hybrid SFM proposed in this paper.

The traditional SFM and hybrid SFM were used for sparse reconstruction of the data set, respectively. The experimental results are shown in Figure 11.

Figure 11a shows the 3D sparse point cloud reconstructed by global SFM, and a total of 822 sparse points are obtained; Figure 11b shows 958 sparse points of 3D sparse point cloud reconstructed by incremental SFM; Figure 11c shows the 3D sparse point cloud reconstructed using the hybrid SFM designed in this paper, and a total of 1554 sparse points are obtained.

**Figure 12:**
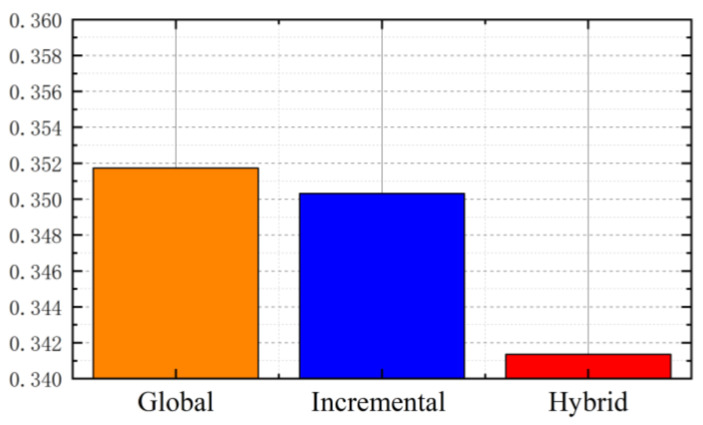
RMSE value of sparse model.

The root mean square of the re-projection error is taken as the standard of reconstruction accuracy. The RMSE value of the sparse model reconstructed by traditional SFM and hybrid SFM is shown in the Figure 12.

Table 1 shows the comparison between the traditional SFM and the hybrid SFM proposed in this paper in terms of reconstruction time, number of point clouds and reconstruction accuracy.

In order to verify the efficiency and accuracy of the improved SFM algorithm compared with the traditional SFM for large data sets, the data set “doll” was tested. The data set has 48 pictures. The traditional SFM and hybrid SFM were used to complete sparse reconstruction, and their reconstruction time, number of point clouds and reconstruction accuracy were compared.

The experimental data of data set “doll” are shown in Table 2.

According to the above experimental data, the number of point clouds of the hybrid SFM model proposed in this paper is more than that of global and incremental SFM; in terms of reconstruction accuracy, the global SFM has the worst accuracy, and the hybrid SFM proposed in this paper is slightly higher than the incremental SFM. In terms of reconstruction time, the global SFM takes the least time, because the global SFM only performs BA optimization once; as the method in this paper needs to perform incremental calculation in the current subset and then perform global optimization, the reconstruction speed improvement effect of hybrid SFM is not significant for small data sets, but for large data sets, compared with the process of triangulation and continuous Ba optimization of successively registered pictures of incremental SFM, hybrid SFM will greatly shorten the reconstruction time. When dealing with large data sets, the reconstruction accuracy of hybrid SFM is about 3.8% higher than that of global SFM, and the reconstruction time is about 21% less than that of incremental SFM. Therefore, the hybrid SFM proposed in this paper has more stability and robustness in sparse point-cloud reconstruction, and is more efficient for large data sets. The experimental results show that the reconstruction accuracy of hybrid SFM is improved compared with traditional SFM. For large data sets, the reconstruction time is also reduced by 21% compared with incremental SFM, which meets the expected requirements and the experimental design is reasonable.

### 4.3. Dense-Reconstruction Experimental Data

Through the dense-reconstruction process mentioned in Section 3.4, the depth map and depth normal vector map corresponding to each picture were generated.

**Figure 13:**
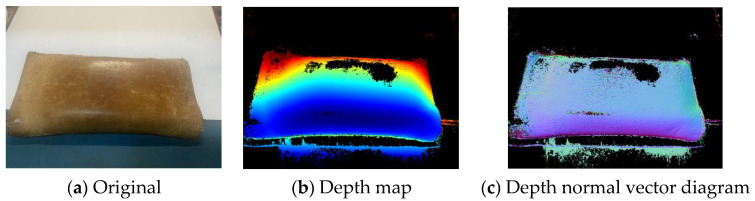
(**a**–**c**) are the original image, depth map and depth normal vector map corresponding to the original map.

Taking the “wallet” dataset as an example, first the depth map corresponding to each image was calculated. The original map, depth map and depth normal vector map are shown in Figure 13. Blue indicates close distance, red indicates far distance, and white indicates that there is no depth value in this area. The black defect is due to the influence of epidermal reflection, but it does not affect the reconstruction effect.

**Figure 14:**
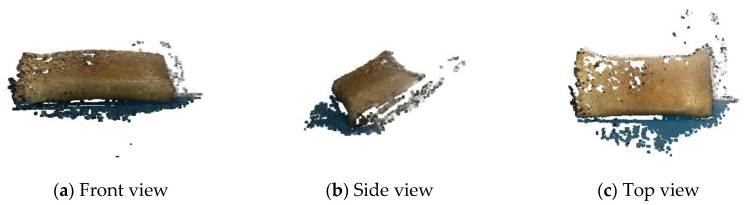
(**a**–**c**) are the dense point-cloud models observed in front view, side view and top view, respectively.

Then, the depth map was fused to obtain the dense point-cloud reconstruction model, as shown in the Figure 14.

According to the dense point-cloud model, a total of 502,683 dense points are obtained, and the contour of the scene can be obtained, but there are still holes and missing areas on the surface, and there is no texture information.

### 4.4. Experimental Data of Surface Reconstruction

**Figure 15:**
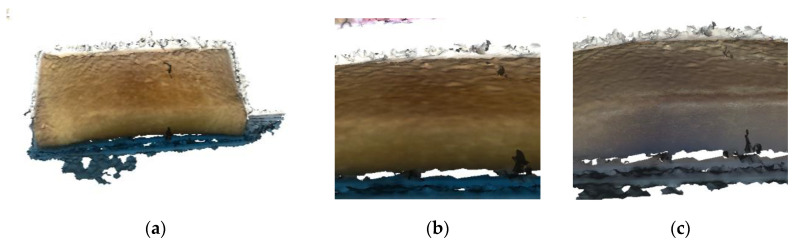
(**a**) Model after surface reconstruction. (**b**) Model before texture image creation. (**c**) Model after texture image creation. The model after surface reconstruction, the model before and after texture creation.

The local enlarged view of the model after surface reconstruction and the model before and after texture creation is shown in Figure 15.

Analysis of experimental results: the surface reconstruction algorithm based on a long symbol distance can clearly reconstruct the surface appearance of the object. Compared with the dense point-cloud model, the surface of the model is complete, smooth and empty, but it cannot reconstruct the texture information; after creating the texture image of the model, the texture information of the object surface can be obtained. At this point, the structural form and surface information of the object “wallet” is basically completely restored.

## 5. Conclusions

This paper designs and implements a set of multi-view 3D reconstruction technology based on SFM; inputs a group of images collected by a camera; extracts, matches and optimizes the feature points of multiple images; designs a hybrid SFM according to the advantages and disadvantages of the traditional SFM algorithm; obtains the external parameters of the camera and the sparse point-cloud model; then obtains the dense point-cloud model by using the MVS algorithm of depth-map fusion; and, finally, completes the surface reconstruction, making the 3D model more realistic.

The experimental results show that the matching and fusion of SIFT and SURF feature results can increase the number of extracted feature points, and the RANSAC algorithm can effectively eliminate false matching; in the sparse reconstruction stage, the hybrid SFM proposed in this paper has the same number of sparse-model point clouds as the incremental SFM. When dealing with large data sets, the reconstruction accuracy error of the hybrid SFM is about 3.8% lower than that of global SFM, and the reconstruction time is about 21% less than that of incremental SFM; in the dense reconstruction, the MVS algorithm of depth-map fusion was used to complete the dense reconstruction, and the contour of the dense model is clear. The surface reconstruction was completed with relevant algorithms to obtain the surface information of the scene, which meets the requirements of the research work.

## Figures and Tables

**Table 1:** Dataset “wallet” experimental data.

	Reconstruction Time (s)	Number of Point Clouds	Model RMSE Value
Global SFM	8	822	0.351732
Incremental SFM	13	958	0.350316
The hybrid SFM proposed in this paper	15	1554	0.341345

**Table 2:** Dataset “doll” experimental data.

	Reconstruction Time (s)	Number of Point Clouds	Model RMSE Value
Global SFM	41	3875	0.404613
Incremental SFM	75	5618	0.402451
The hybrid SFM proposed in this paper	59	5762	0.3886483
